# Whole genome analysis and antimicrobial resistance of *Neisseria gonorrhoeae* isolates from Ghana

**DOI:** 10.3389/fmicb.2023.1163450

**Published:** 2023-06-29

**Authors:** Bright Agbodzi, Samuel Duodu, Helena Dela, Selassie Kumordjie, Clara Yeboah, Eric Behene, Karen Ocansey, Jennifer N. Yanney, George Boateng-Sarfo, Samuel Kojo Kwofie, Beverly Egyir, Sophie M. Colston, Hugo V. Miranda, Chaselynn Watters, Terrel Sanders, Anne T. Fox, Andrew G. Letizia, Michael R. Wiley, Naiki Attram

**Affiliations:** ^1^Naval Medical Research Unit No. 3, Accra, Ghana; ^2^West African Centre for Cell Biology of Infectious Pathogens, College of Basic and Applied Sciences, University of Ghana, Accra, Ghana; ^3^Department of Biochemistry, Cell and Molecular Biology, University of Ghana, Accra, Ghana; ^4^Biomedical Engineering Department, School of Engineering Sciences, University of Ghana, Accra, Ghana; ^5^Bacteriology Department, Noguchi Memorial Institute for Medical Research, University of Ghana, Accra, Ghana; ^6^Center for Bio/Molecular Science and Engineering, Naval Research Laboratory, Washington, DC, United States; ^7^Nebraska Medical Center, Omaha, NE, United States

**Keywords:** *Neisseria gonorrhoeae*, antimicrobial resistance, Ghana, whole genome sequencing, gonorrhea, core genome MLST

## Abstract

**Introduction:**

Gonorrhoea is a major public health concern. With the global emergence and spread of resistance to last-line antibiotic treatment options, gonorrhoea threatens to be untreatable in the future. Therefore, this study performed whole genome characterization of *Neisseria gonorrhoeae* collected in Ghana to identify lineages of circulating strains as well as their phenotypic and genotypic antimicrobial resistance (AMR) profiles.

**Methods:**

Whole genome sequencing (WGS) was performed on 56 isolates using both the Oxford Nanopore MinION and Illumina MiSeq sequencing platforms. The Comprehensive Antimicrobial Resistance Database (CARD) and PUBMLST.org/neisseria databases were used to catalogue chromosomal and plasmid genes implicated in AMR. The core genome multi-locus sequence typing (cgMLST) approach was used for comparative genomics analysis.

**Results and Discussion:**

*In vitro* resistance measured by the E-test method revealed 100%, 91.0% and 85.7% resistance to tetracycline, penicillin and ciprofloxacin, respectively. A total of 22 sequence types (STs) were identified by multilocus sequence typing (MLST), with ST-14422 (*n* = 10), ST-1927 (*n* = 8) and ST-11210 (*n* = 7) being the most prevalent. Six novel STs were also identified (ST-15634, 15636-15639 and 15641). All isolates harboured chromosomal AMR determinants that confer resistance to beta-lactam antimicrobials and tetracycline. A single cefixime-resistant strain, that belongs to *N. gonorrhoeae* multiantigen sequence type (NG-MAST) ST1407, a type associated with widespread cephalosporin resistance was identified. *Neisseria gonorrhoeae* Sequence Typing for Antimicrobial Resistance (NG-STAR), identified 29 unique sequence types, with ST-464 (*n* = 8) and the novel ST-3366 (*n* = 8) being the most prevalent. Notably, 20 of the 29 STs were novel, indicative of the unique nature of molecular AMR determinants in the Ghanaian strains. Plasmids were highly prevalent: pTetM and p*bla*TEM were found in 96% and 92% of isolates, respectively. The TEM-135 allele, which is an amino acid change away from producing a stable extended-spectrum β-lactamase that could result in complete cephalosporin resistance, was identified in 28.5% of the isolates. Using WGS, we characterized *N. gonorrhoeae* strains from Ghana, giving a snapshot of the current state of gonococcal AMR in the country and highlighting the need for constant genomic surveillance.

## Introduction

Gonorrhea is a sexually transmitted infection (STI) caused by the obligate human pathogen *Neisseria gonorrhoeae*. The disease is among the most reported STIs and remains a global health issue with high morbidity that translates into low productivity and economic losses (Wi et al., 2017; Rowley et al., 2019). Antimicrobial resistance (AMR) remains the main concern with gonorrhea treatment and management. *N. gonorrhoeae* has developed or acquired nearly all known physiological mechanisms of AMR to all antimicrobials recommended for treatment (Tapsall, 2001; Ohnishi et al., 2011; Unemo et al., 2014; Unemo and Shafer, 2014). These processes have resulted in multidrug resistance (MDR) and treatment failure of antibiotics such as sulphonamides, penicillins, tetracyclines, macrolides, fluoroquinolones, and extended-spectrum cephalosporins (ESCs) (Unemo, 2015; Wi et al., 2017). The cumulative effects of multiple AMR determinants and their complex interactions are essential in the development of clinically significant AMR levels in *N. gonorrhoeae* (Tapsall, 2001, 2006; Unemo and Shafer, 2014).

Considerable progress has been made in surveilling the emergence and spread of gonococcal AMR in Europe and North America, which may be attributable to the impact of next generation sequencing (NGS) (Unemo and Shafer, 2014; Allen and Melano, 2016; Golparian et al., 2020). However, the situation is far from being under control in Africa (Wi et al., 2017). For instance, only 7 African countries are active contributory members to the Gonococcal Antibiotic Resistance Surveillance Programme (GASP) (Unemo et al., 2019). Countries like Ivory Coast, Uganda, Kenya, Zimbabwe and South Africa (Kularatne et al., 2016; Omolo et al., 2017; Latif et al., 2018; Yéo et al., 2019; Workneh et al., 2020) have only recently established surveillance programs based on World Health Organization (WHO) recommendations. Regarding gonococcal whole genome sequencing (WGS), there are a limited number of studies in the African region, primarily occurring in Kenya and South Africa (Lewis et al., 2013; Cehovin et al., 2018; Maduna et al., 2020; Juma et al., 2021; Mitchev et al., 2021). The limited data from the African region creates a blind spot in the world’s surveillance efforts.

In Ghana, there are no established gonococcal AMR surveillance programs and there have been few studies on AMR levels in *N. gonorrhoeae* in recent years (Donkor and Newman, 2011; Duplessis et al., 2013; Attram et al., 2019; Dela et al., 2019). In the most recent reported *N. gonorrhoeae* study in Ghana, using *N. gonorrhoeae* multiantigen sequence typing (NG-MAST), Attram and colleagues identified one isolate with reduced susceptibility to cefixime, which raised concern for the possible emergence of untreatable strains of *N. gonorrhoeae* and the need for constant genomic surveillance (Attram et al., 2019). Notably, there currently is no *N. gonorrhoeae* whole genome surveillance data from the West African region, including Ghana, thereby, limiting our knowledge about the genomic epidemiology and the repertoire of genomic AMR determinants of gonococci in the region.

Surveillance efforts provide critical data that could be used to implement infection countermeasures and provide information for national government strategy development. Therefore, this study sought to address the knowledge gap in gonococcal AMR in Ghana. Here, we describe the genomic epidemiology and AMR characteristics of 56 Ghanaian gonococcal isolates collected between 2012 and 2019 and how they compare with isolates from other regions.

## Methodology

This was a retrospective study analyzing archived *N. gonorrhoeae* isolates collected between 2012 and 2019 during a gonococcal AMR surveillance study. Previously cultured isolates with known antimicrobial susceptibility testing data were retrieved from storage in tryptic soy broth (TSB) with 20% glycerol at −80°C and revived on GC agar with 1% IsoVitaleX and 1% heme. Isolates were reconfirmed using catalase and oxidase testing as well as Gram staining prior to DNA extraction.

## DNA extraction and whole genome sequencing

Genomic DNA was extracted from pure freshly grown or archived *N. gonorrhoeae* isolates using the QIAamp® DNeasy Ultraclean Microbial kit (Qiagen, Hilden, Germany) following manufacturer’s procedures. Illumina libraries were prepared using the Kapa HyperPlus Library Prep Kit (Kapa Biosystems, Massachusetts, United States) per manufacturer’s instructions. Short-read sequencing was performed on the Illumina MiSeq platform using V3 reagent kit (Illumina, USA) generating 2 × 300 base paired-end reads. Sequencing libraries for Oxford Nanopore Technology (ONT) were prepared using the Rapid Barcoding kit (SQK-RBK004) (Oxford Nanopore Technologies, United Kingdom) and sequenced on FLO-MIN106 R9.4.1 flow cells/Mk-1B MinION sequencer following the manufacturers protocol. Sequencing was performed at the Noguchi Memorial Institute for Medical Research (NMIMR) in Ghana.

## Whole genome sequence analysis

Illumina reads were base-called and demultiplexed on the Miseq. The raw fastq files generated from the MiSeq were quality filtered to Phred score ≥ 20, filtered for minimum read length of 50 bp, and adapter trimmed using BBDuk Trimmer v 1. Read quality was confirmed using the FastQC tool.^1^ For ONT, raw reads were base-called and demultiplexed in real-time via the MinKNOW software (Oxford Nanopore Technologies, Oxford, UK). Only reads designated as pass were included for further processing. The pass reads were trimmed off adapter sequences using Porechop (v0.2.1 available from: https://github.com/rrwick/Porechop). Nanofilt (v.1.0.5, available from: https://github.com/wdecoster/nanofilt) was used to quality-filter trimmed reads to remove reads with average quality <9 and length shorter than 500 bp. Quality filtered and trimmed raw fastq files from both sequencing technologies described above were used as input files to perform Illumina-ONT hybrid assemblies using SPAdes v 3.11.1 in ‘careful’ mode.

## Genome annotation and typing

The contigs generated from the hybrid assembly were submitted to PubMLST (PUBMLST.org/neisseria) for annotation. The BIGSdb software at PubMLST automatically identifies and annotates defined loci with ≥98% sequence identity to alleles in the database. The BIGSdb annotation enabled assignment of multi-locus sequence typing (MLST) sequence types (STs), *Neisseria gonorrhoeae* Multiantigen Sequence Typing (NG-MAST), *Neisseria gonorrhoeae* Sequence Typing for Antimicrobial Resistance (NG-STAR) types, identification of resistance markers, plasmids, the presence of gonococcal genomic islands, etc. The novel sequence types were submitted to the curators of the databases of the various typing schemes for assignment of new types.

## Annotation of AMR determinants

The data generated from the assemblies were submitted to PubMLST.org and the Comprehensive Antimicrobial Resistance Database (CARD)^2^ for annotation of AMR determinants. For CARD annotations, the Resistance Gene Identifier^3^ tool was used. The results designated in the “perfect” and “strict” categories were considered for evaluation. The presence of point mutations that were implicated in resistance were manually confirmed by performing alignments in Geneious Prime, v.2020.2.4.

## Comparative genomics

To understand how the strains from Ghana compare to each other and to those from different parts of the world, whole genome phylogeny was constructed based on concatenated core genome loci (core genome MLST, cgMLST). Using the Genome Comparator tool on the BIGSdb platform at: https://pubmlst.org, 56 gonococcal genomes were compared alongside 3 reference genomes, 14 WHO AMR reference strains and 70 other genomes originating from Africa, United States, United Kingdom, and China. The sequence data of these isolates were retrieved from PubMLST.org.^4^ Two groups of isolates were selected for the analysis, the first was made up of isolates that belonged to MLST types present in our isolates (ST-11366, ST-1588, ST-1893, ST-1599, ST-1603, ST-1931, ST-1579, ST-11422, ST-1583, and ST-1596). The second group of isolates were those of MLST types that have been associated with highly resistant clones that are internationally disseminated, but were not present in our isolates (ST-1901, ST-9363, ST-7363 and ST-7367; Unemo et al., 2012, 2016; Lefebvre et al., 2018; Lee et al., 2019). The *N. gonorrhoeae* cgMLST v1.0 scheme was implemented with a core genome threshold of loci present in 95% of all genomes. The generated core genome alignments were used to construct maximum likelihood phylogenies using RaxML v8.2.11 under the General Time Reversible (*GTR*) GAMMA nucleotide substitution model, and computing 1,000 bootstrap replicates. The resultant trees were visualized and annotated in FigTree v1.4.4.

## Results

### Data availability

A total of 56 gonococcal isolates were sequenced using both the Illumina MiSeq and the Oxford Nanopore MinION platforms. A summary of sequencing and assembly metrics is presented in Supplementary Table S2. The whole genome assemblies were deposited at DDBJ/ENA/GenBank under the BioProject ID PRJNA788433; accession numbers (JAJQRH000000000-JAJQTK000000000) and IDs 95771 to 95821 & 111732 to 111736 at PubMLST.org.

### Antimicrobial susceptibility testing

*In vitro* resistance measured by the E-test, revealed that 100, 91.0 and 85.7% of the isolates were resistant to tetracycline, penicillin and ciprofloxacin, respectively. The minimum inhibitory concentrations (MICs) for the resistant isolates ranged from 2 to 192 μg/mL for tetracycline, 3 to >32 μg/mL for penicillin and 3 to >256 μg/mL for ciprofloxacin. One isolate with reduced susceptibility to cefixime was recorded (MIC: 0.75 μg/mL). MIC ranges of 0.002–0.016 μg/mL and 0.016–0.75 μg/mL for ceftriaxone and cefixime, respectively, were recorded. No azithromycin resistant isolate was recorded. MIC range of 0.064–0.75 μg/mL was recorded for azithromycin. No spectinomycin resistance was recorded. The results of the antimicrobial susceptibility testing of all isolates are summarized in Table 1.

**Table 1:** Antimicrobial susceptibility profiles of the 56 *Neisseria gonorrhoeae* isolates from Ghana.

Antimicrobial	No. (%) of isolates		
	S	I	R
Penicillin	1 (1.8)	4 (7.1)	51 (91.0)
Tetracycline	0 (0)	0 (0)	56 (100)
Ciprofloxacin	5 (8.9)	3 (5.3)	48 (85.7)
Spectinomycin	56 (100)	0 (0)	0 (0)
Azithromycin*	56 (100)	0 (0)	0 (0)
Ceftriaxone*	56 (100)	0 (0)	0 (0)
Cefixime*	55 (98.2)	0 (0)	1 (1.8)

CLSI 2018 breakpoints were used for penicillin, tetracycline, spectinomycin and ciprofloxacin. GISP breakpoints (alert values) were used for azithromycin, cefixime, and ceftriaxone for which CLSI resistance breakpoints are not established (Supplementary Table S1).

*No resistance breakpoint available (R represents reduced-susceptibility).

S, susceptible; I, Intermediate; R, resistant/reduced-susceptibility.

CLSI, Clinical and Laboratory Standards Institute.

GISP, Gonococcal Isolate Surveillance Project.

### Molecular epidemiology and AMR typing analysis

A total of 22 sequence types (STs) were identified by MLST, with ST-14422 (*n* = 10), ST-1927 (*n* = 8) and ST-11210 (*n* = 7) being the most prevalent. Six novel STs were also identified and submitted for the assignment of new sequence types (ST-15634, 15636–15639 and 15641). NG-MAST produced 25 sequence types with ST8948 (*n* = 7), ST12791 (*n* = 6) and ST10251 (*n* = 4) being the most prevalent. Notably, one ST1407, which has been associated with elevated MICs in cephalosporins, was identified. Thirteen novel STs were also identified and submitted for the assignment of new sequence types (ST-19707-19719). NG-STAR identified 29 sequence types with ST-464 (*n* = 8) and the novel ST-3366 (*n* = 8) being the most prevalent. Notably, 20 of the 29 STs were novel. The novel sequence types were submitted for assignment of new sequence types (ST-3352-3372). The results of the molecular epidemiology and AMR typing analysis are summarized in Table 2.

**Table 2:** Molecular epidemiology and AMR typing of the 56 *N. gonorrhoeae* isolates from Ghana.

Molecular epidemiological typing				AMR typing	
MLST		NG-MAST		NG-STAR	
ST	No. of isolates	ST	No. of isolates	ST	No. of isolates
14422	10	8948	7	464	8
1927	8	12791	6	**3366**	8
11210	7	10251	4	**3361**	5
1588	5	16222	2	1215	5
1603	4	3370	2	567	3
**15641**	3	355	2	**3363**	2
**15639**	2	16226	2	**3352**	2
11365	2	16227	2	**3359**	2
1596	2	20346	1	**3353**	1
**15638**	1	16221	1	**3354**	1
**15637**	1	1737	1	**3355**	1
**15636**	1	16217	1	**3356**	1
**15634**	1	211	1	**3357**	1
1591	1	16218	1	**3358**	1
1583	1	16219	1	**3360**	1
11241	1	3178	1	**3362**	1
1579	1	1407	1	**3364**	1
1893	1	16225	1	**3365**	1
1931	1	16228	1	**3367**	1
14789	1	16231	1	**3368**	1
13766	1	9523	1	**3370**	1
11976	1	16232	1	**3371**	1
		2025	1	**3372**	1
		15597	1	438	1
		**19707–19719**	(1 of each)	157	1
				567	1
				308	1
				893	1
				891	1
Novel	6	Novel	13	Novel	20
Total	22	Total	36	Total	29

Bold STs are unique to this study.

### Comparative genomics

**Figure 1:**
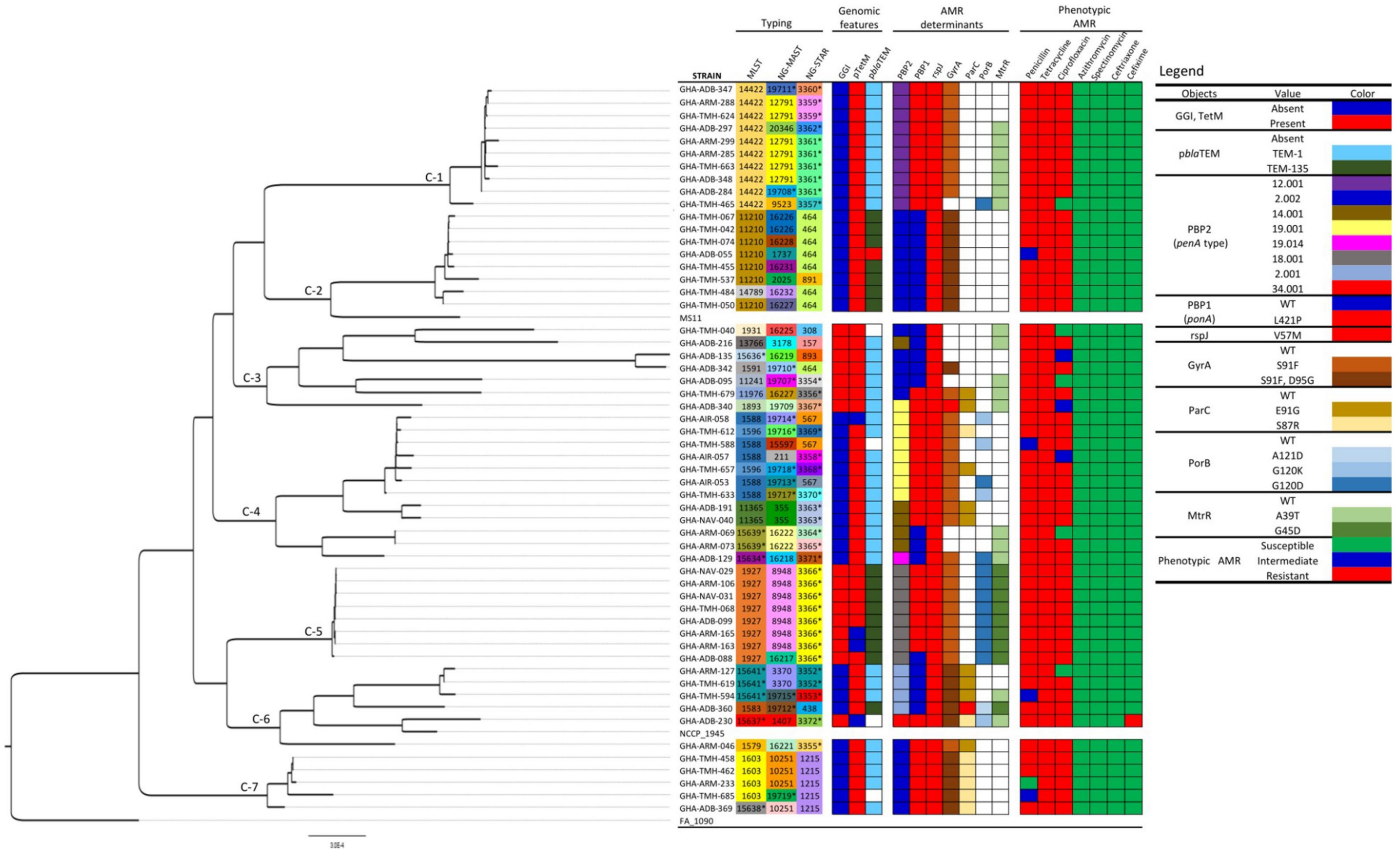
Phylogeny, genomic epidemiological typing, genotypic, and phenotypic AMR characteristics of *Neisseria gonorrhoeae* strains analyzed in this study. The seven distinct clades observed are labeled C1-C7. Reference strains FA 1090, NCCP 1945 and MS11 were included for context. Sequence types unique to this study are highlighted with asterisks (*). The phylogeny was inferred using concatenated core genome loci extracted using Genome Comparator on BIGSdb platform at PubMLST.org. The phylogenetic tree was constructed using RaXML v8.2.11 and visualized in FigTree v1.4.4.

**Figure 2:**
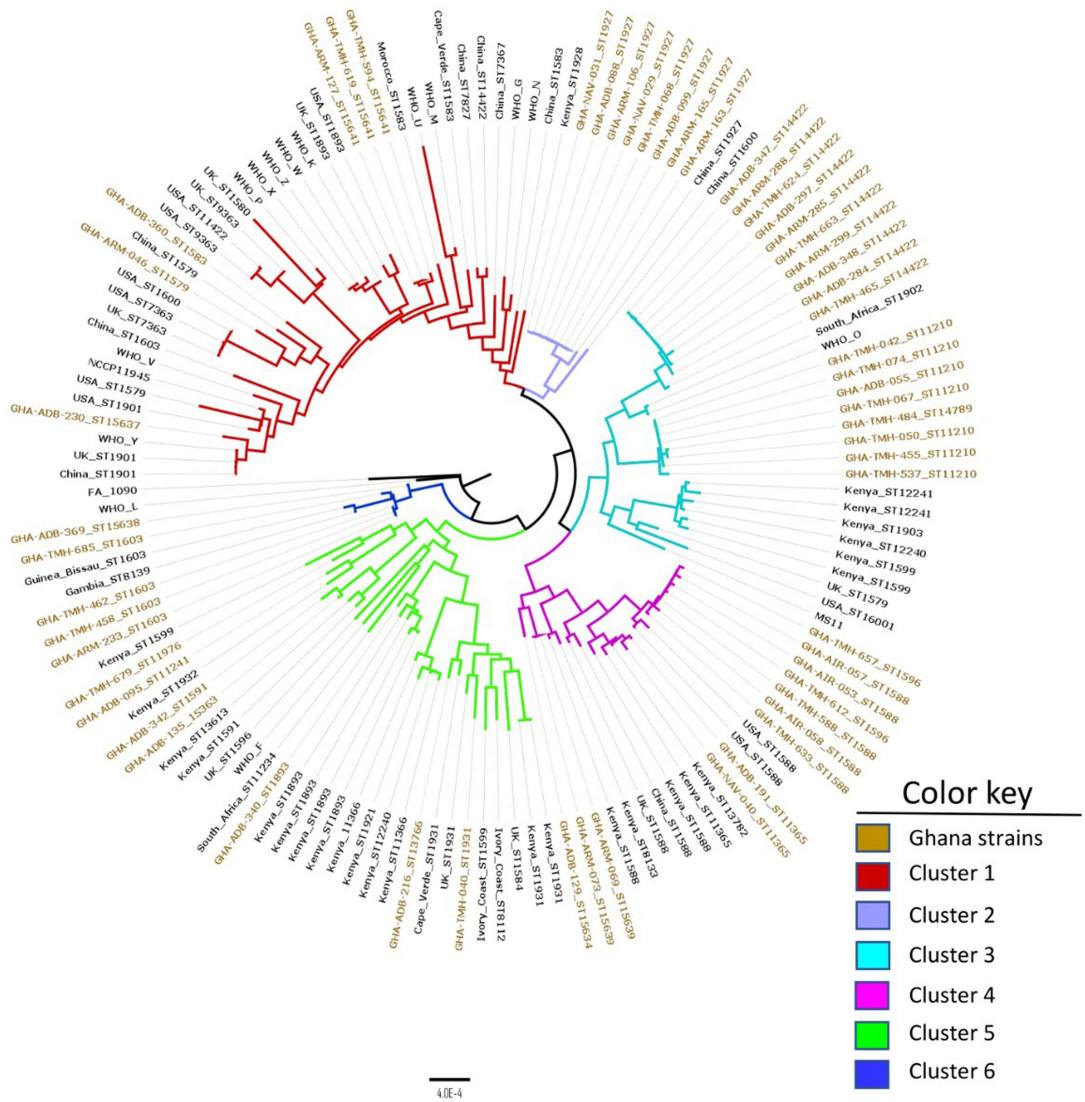
Core genome genealogy of *N. gonorrhoeae* strains from Ghana compared to strains obtained from different parts of the world, as well as WHO AMR reference strains. The strains are labeled with their MLST STs and country of origin. The phylogeny was inferred using concatenated core genome loci extracted using Genome Comparator on BIGSdb platform at PubMLST.org. The phylogenetic tree was constructed using RaXML v8.2.11 and visualized in FigTree v1.4.4.

Using the cgMLST approach, seven distinct clades were observed when the 56 Ghanaian isolates were analyzed by phylogeny. The genomic epidemiology results, genomic characteristics, and the genotypic and phenotypic AMR characteristics were used to annotate the phylogenetic tree (Figure 1). Furthermore, comparing the Ghanaian isolates to some international strains revealed six main clusters (Figure 2). In the highly diverse cluster 1, the only ESC resistant strain from this study with the novel ST-15637 grouped with the internationally disseminated ESC resistant strain of the ST-1901 from the UK, the US, and China. Also present in this cluster were Ghanaian strains of the STs 1579, 1583, and the novel 15641. Cluster 2 was made up of a monophyletic clade of ST-1927 Ghanaian isolates, which clustered with ST-1927 and ST-1600 strains from China. Cluster 3 consisted of three subclades. The first was a monophyletic clade of ST-14422 isolates from Ghana related to the WHO O strain and an ST-1902 strain from South Africa. The second clade contained Ghanaian isolates mainly of the ST-11210; while the other clade, contained isolates of different STs originating from Kenya, the US, and the UK. Cluster 4 consisted of Ghanaian isolates mainly of the ST-1588, along with strains from Kenya, China, the UK, and the US. Other STs of Ghanaian origin were also present in cluster 4, including 1596 and 11365. Cluster 5 consisted of a highly diverse collection of isolates with different STs. The Ghanaian isolates with internationally disseminated STs in this cluster (ST-1893 and ST-1931) were related to isolates from Kenya and the UK. Isolates in cluster 6 consisted mainly of ST-1603 Ghanaian strains that were related to the WHO L strain, a Chinese ST-1600 strain, and two other strains from Africa (ST-1603; Guinea Bissau and ST-8139; Gambia).

**Figure 3:**
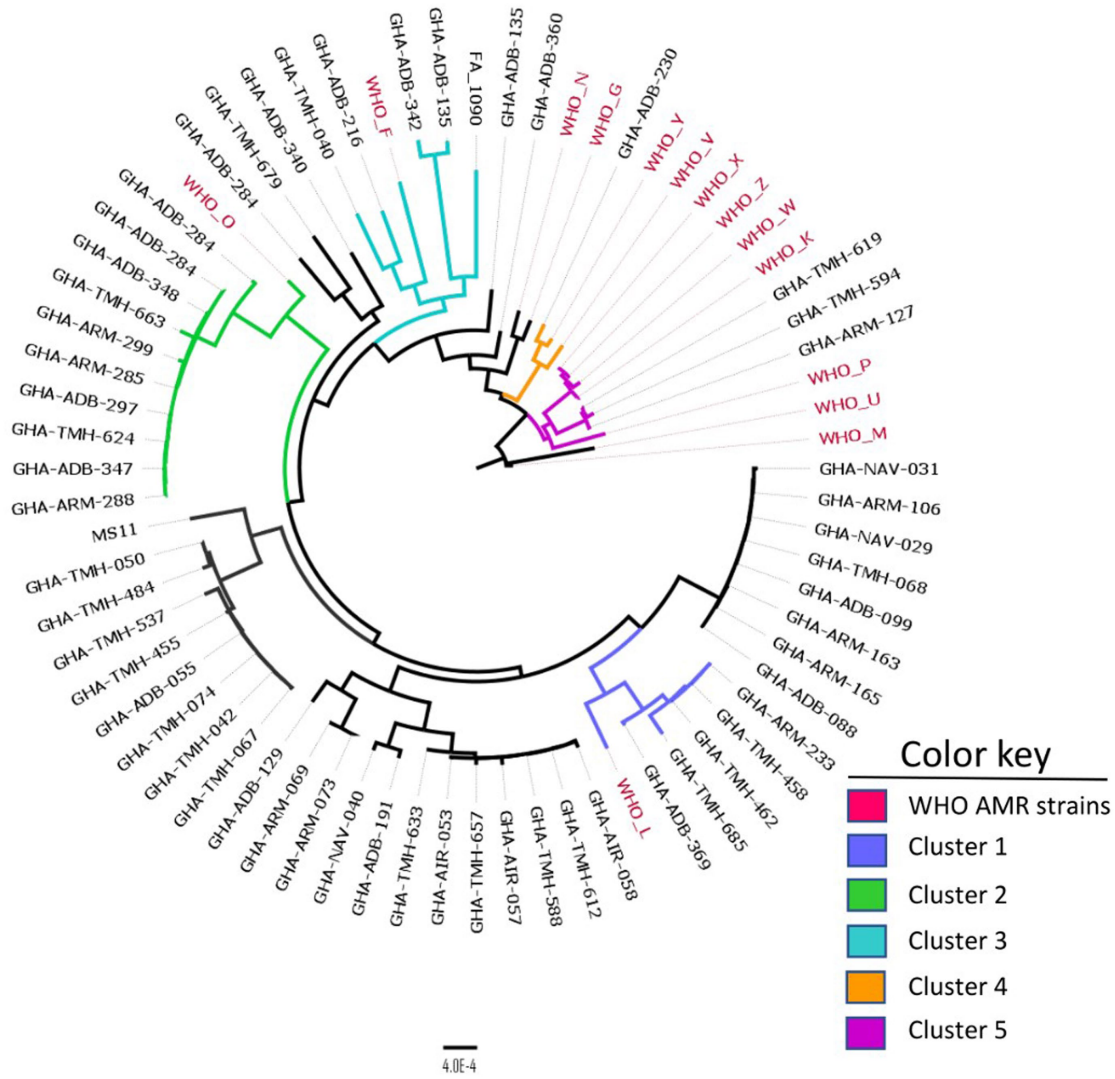
Core genome MLST clustering of the 56 isolates alongside the 14 WHO AMR strains. Five distinct clusters were obtained with each cluster defined by a color key represented above. The phylogeny was inferred using concatenated core genome loci extracted using Genome Comparator on BIGSdb platform at PubMLST.org. The phylogenetic tree was constructed using RaXML v8.2.11 and visualized in FigTree v1.4.4.

A comparison between Ghanaian strains and the 14 WHO AMR reference strains using cgMLST revealed five distinct clusters that contained Ghanaian and WHO strains (Figure 3). In cluster 1, the Ghanaian strains grouped with WHO L, a ceftriaxone resistant strain belonging to MLST ST-1590; however, the Ghanaian strains in this cluster belonged to MLST ST-1603. Cluster 2 isolates were closely related to WHO O strain, an ESC susceptible strain belonging to MLST ST-1902. However, all the Ghanaian strains in this cluster belonged to MLST ST-14422. Isolates in cluster 3 were related to WHO F, a strain which is susceptible to all the commonly used antibiotics in gonorrhea treatment (Unemo et al., 2016). In cluster 5, the only ESC (cefixime) resistant strain from the current study clustered with WHO Y and WHO V strains, which are ESC resistant strains (Unemo et al., 2016). In the last cluster, 6, the Ghanaian isolates grouped with WHO strains X, Z, W, and K, all of which are ESC resistant strains.

### Genotypic AMR determinants

All isolates harbored chromosomal AMR determinants that confer resistance to beta-lactam antimicrobials and tetracycline. Isolates exhibited one of the 4 different *penA* AA substitution patterns (P) in (P1: F504L, A510V, A516G; P2: A501T, G542S, F504L, A510V, A516G; P3: F504L, A510V, A516G, P551S; and P4: F504L, A510V). Seven different *penA* alleles were identified, with non-mosaic allele II being the most prevalent. The only mosaic *penA* allele was found in the isolate with reduced susceptibility to cefixime. Amino acid substitution L421P in *ponA* (*ponA1* allele), which is also associated with resistance in beta-lactams, was identified in 35 (62.5%) of the isolates. Mutations in *porB,* which are associated with resistance to tetracycline and beta-lactams, were identified in 16 (28.5%) isolates. Each of the three characterized mutations in *porB* (G120D, G120K, and A121D) were present. Fluoroquinolone resistance determinants present in the *gyrA* and *parC* gene segments were identified. Mutations in *gyrA* were present in 51 (91.0%) of the isolates. The S91F and D95G were present in 49 (87.5%) and 20 (36.0%) isolates, respectively, while 20 (35.7%) had both mutations present. Mutations in *parC,* the other fluoroquinolone resistance marker, were identified in 16 (28.5%) of the isolates, with both the E91G (16.0%) and S87R (12.5%) mutations present in 9 and 7 isolates, respectively. The *mtrR* mutations A39T 17 (30.3%) or G45D 9 (16.0%), which have been associated with azithromycin and beta-lactam resistance, were also detected. All 56 isolates contained the point mutation V57M in the *rpsJ* gene, which has been associated with high-level chromosomally mediated tetracycline resistance. The amino acid substitution G70D was present in the *rpld* and *macB* resistance markers associated with macrolide resistance. The *rpld* and *macB* mutations were present in 4 (7.1%) and 13 (23.2%) of isolates, respectively. A catalog of all genotypic AMR determinants is presented in Table 3. Concordance between AMR genotypes and phenotypes were above 70 % for penicillin, tetracycline and ciprofloxacin, while very low concordance was recorded for azithromycin and the cephalosporins (Table 4).

**Table 3:** Resistance-Associated mutations/gene(s) in the 56 *N. gonorrhoeae* isolates from Ghana.

Gene	AMR-Associated amino acid substitutions	Prevalence N (%)	Antibiotic group(s)
PBP2	A501T	8 (14)	Beta-lactams: Penicillins and Cephalosporins
	G542S	8 (14)	
	F504L	56 (100)	
	A510V	56 (100)	
	A516G	55 (98)	
	P551S	10 (18)	
PBP1	L421P	35 (62.5)	
gyrA	S91F	49 (87.5)	Fluoroquinolones: Ciprofloxacin
	D95G	20 (36)	
parC	E91G	9 (16)	
	S87R	7 (12.5)	
mtrR	A39T	17 (30)	Beta-lactams & Azithromycin
	G45D	9 (16)	
penB	G120D	11 (20)	Beta-lactams and Tetracyclines
	G120K	4 (7)	
	A121D	1 (2)	
rpsJ	V57M	56 (100)	Tetracyclines
rpld	G70D	4 (7)	Macrolides
macB	Not relevant	13 (23)	
blaTEM	Not relevant	52(92.8)	Beta-lactams
tetM	Not relevant	52(92.8)	Tetracyclines

**Table 4:** Concordance between AMR phenotypes and genotypes in the 56 *N. gonorrhoeae* isolates from Ghana.

Antibiotic	Phenotype–Genotype concordance
Penicillin	51/56 (91.0%)
Tetracycline	56/56 (100%)
Ciprofloxacin	41/56 (73.2%)
Azithromycin	0/56 (0)
Ceftriaxone	0/56 (0)
Cefixime	1/56 (1.8%)

### Plasmid-mediated AMR

The pTetM conjugative plasmid was present in 54 (96%) of the isolates. However, the *tetM* determinant that is responsible for high-level plasmid-mediated tetracycline resistance was present in 52 (92.8%) of the isolates. Thirteen Dutch (allele 1) and 41 American (allele 2) types were present. Four of the American type plasmids were highly divergent, suggesting a different ancestry. The non-conjugative plasmid p*bla*TEM was present in 52 (92.8%) of the isolates. Thirty-six of the *bla*TEM plasmids were of the TEM-1 allele type while the remaining 16 were of the TEM-135 allele type. The prevalence of p*bla*TEM plasmid types classified by geographical origin were as follows: Johannesburg (28), Australian (16), African (6), Asian (1) and a highly divergent, possibly novel, plasmid type.

## Discussion

In this work, we used WGS to analyze the genealogy and AMR of gonococcal isolates from Ghana, the first study of its kind in West Africa. These data build upon previous work (Attram et al., 2019) by further describing genomic epidemiology and AMR gonococcal isolates from Ghana. From the WGS perspective, we demonstrate that gonococci from Ghana constitute a unique genomic pool with distinct AMR characteristics evident in the number of novel STs identified.

Overall, the MLST data revealed that the isolates collected in this study were diverse with 22 different STs of which six were novel. The most prevalent STs included ST-14422, ST-1927, ST-11210 and ST-1588. Of these STs, ST-1588, is the most globally distributed having been previously reported in Africa (Harrison et al., 2016; Cehovin et al., 2018; Lee et al., 2019), Europe, America (da Costa-Lourenço et al., 2017) and Asia (Nakayama et al., 2012). ST-1927 is geographically restricted and mostly identified in Asia (Liu et al., 2018; Yan et al., 2019) and Russia (Ilina et al., 2010). ST-14422 and ST-11210 are less common with ST-14422 traced back to China and the US as sequence origin^5^ (Gernert et al., 2020). The remaining 12 STs were highly diverse and globally disseminated. Comparative genomics analysis between the Ghanaian strains and strains of different geographical origin (Figure 2) highlighted the unique nature of the Ghanaian strains and the need for constant monitoring. NG-MAST characterization of the isolates resulted in identification of 25 STs, including 13 that were found to be unique. Of note is the single ST1407 strain that contains the mosaic *penA* gene of the allele type 34.001 associated with widespread cephalosporin resistance (Unemo et al., 2012; Sánchez-Busó et al., 2019). Although ST1407 isolates usually belong to MLST ST-7363 and ST-1901 (Shimuta et al., 2015), the ST1407 isolate recovered from this study had a novel MLST ST (ST-15637), suggesting the possibility of clonal diversity. The comparative genomics analysis using the cgMLST approach revealed that most of the Ghanaian isolates formed compact clusters and were separated from non-Ghanaian strains. This suggests that Ghanaian strains could be endemic to the region and are locally transmitted.

NG-STAR characterization identified 29 distinct STs. Interestingly, 20 of these STs were novel, indicating the unique nature of the assembly of genotypic AMR determinants and their possible phenotypic manifestations. The origin of the most prevalent previously described ST, ST-464, could not be traced, while ST-3366 that appears at the same frequency was unique to this study. Of the seven loci, the 23S rRNA gene associated with azithromycin resistance was the most conserved, with all isolates exhibiting the wild-type allele 100. On the other hand, the *mtrR* gene associated with beta-lactam and macrolide resistance had the most diverse array of 15 alleles present in the isolates. The unique genotypic AMR characteristics of the Ghanaian strains were also evident in the phylogeny between the WHO AMR reference strains since many of our isolates barely clustered with the WHO AMR reference strains (Figure 3).

In this study, the only multi-drug resistant strain (MDR), GHA-ADB-230, belonged to MLST 15637 and NG-MAST 1407 and was collected in 2015. This is the second mention of cephalosporin resistance in Africa after the first reported case of treatment failure to an ESC in South Africa (Lewis et al., 2013; Attram et al., 2019). The South African strains however, belonged to different sequence types (MLST 1901 and NG-MAST 4822). The GHA-ADB-230 strain possessed the F504L, A510V, N512Y and G545S substitutions, which are present in well-characterized ESC resistant strains (Ito et al., 2005; Thakur et al., 2014; Unemo et al., 2016; Thakur et al., 2017). Unsurprisingly, it was the only isolate with an elevated MIC to an ESC, showing a cefixime MIC of 0.75 μg/mL. This isolate also contained the A39T and G120K mutation in *mtrR* and *porB,* respectively, which have been associated with ESC resistance (Thakur et al., 2014). The identification of an isolate with reduced susceptibility to an ESC is a cause for concern considering the risk of possible spread. Although contact tracing was not done to halt the spread of this strain, it has fortunately remained the only ESC resistant isolate to date. This finding cannot be overstated considering the deficiencies in the Ghanaian surveillance system. All the remaining isolates harbored at least three amino-acid substitutions implicated in reduced susceptibility to ESCs (Whiley et al., 2007; Liao et al., 2011; Thakur et al., 2014, 2017). Although none of the isolates expressed resistance phenotypically, they harbored mutations, that are associated with resistance to the ESCs. All these manifestations point to the looming possibility of emergence of resistance to ESC and the need for action. With the gradual development of resistance to ESCs, dual therapy (e.g., azithromycin-ceftriaxone; azithromycin-gentamycin) remains one of the most promising treatment options in the foreseeable future (Unemo and Workowski, 2018; Rob et al., 2020). However, it will be interesting to know the status of azithromycin resistance in the near future after its widespread use during the COVID-19 pandemic. Phenotypic resistance to penicillin, tetracycline and ciprofloxacin remains extremely high, pointing to the need for continual antimicrobial stewardship to limit public access to these readily available antimicrobials. Contrary to the observation by Harrison et al. (2016), the presence of a gonococcal genetic island (GGI) in the Ghanaian isolates was not associated with AMR. Concordance between AMR genotypes and phenotypes were above 70% for penicillin, tetracycline and ciprofloxacin; similar to a previous report (Harrison et al., 2016) while discrepancies were recorded for azithromycin and the cephalosporins. The discrepancies observed could be attributed to the lag between a genetic mutation and the manifestation of the associated phenotype (Carballo-Pacheco et al., 2020). Plasmids have played an integral role in emergence and spread of high-level resistance to penicillin and tetracycline in gonococcus (Nakayama et al., 2012; Unemo et al., 2016). The high prevalence (96%) of the pTetM conjugative plasmid observed was similar to that observed in Kenya (97%) and South Africa where prevalence between 73–84% have been recorded (Fayemiwo et al., 2011; Cehovin et al., 2018; Maduna et al., 2020; Mitchev et al., 2021). The p*bla*TEM plasmid plays a key role in resistance to beta-lactam antibiotics including benzyl penicillin, ampicillin and cephaloridine (Cehovin et al., 2017). Both TEM-1 and TEM −135 allele types were detected in this study. The identification of TEM-135 in some isolates (28.5%) is of concern because further changes to specific amino-acids might lead to the production of a stable extended-spectrum β-lactamase, which could result in complete cephalosporin resistance (Nakayama et al., 2012). Although the TEM-135 has been reported to be carried by the Rio/Toronto and Asian plasmids predominantly (Muhammad et al., 2014; Yan et al., 2019), all TEM-135 alleles identified in this study were carried by the Australian plasmid type. The observed MICs greater than the E-test measurable limit (32 μg/mL) for the *bla*TEM-135 possessing strains is in agreement with the observation by Yan et al. (2019) who reported that *bla*TEM-135 strains in their study exhibited the highest penicillinase activity and MICs.

There are some potential limitations to this study. First, the retrospective nature of the study limits assessing temporal relationships and associations in the data. Second, the samples analyzed were from only two regions in southern Ghana; therefore, the results may not be a true reflection of the situation across the country. Third, the small sample size made it difficult to draw associations between observations. Despite these limitations, this study was the first to apply WGS to investigate gonococcal genomic epidemiology and AMR in Ghana and West Africa and has highlighted the potential of using WGS to monitor gonococcal AMR.

The future of gonorrhea management and treatment is hinged on the development of effective regional and global surveillance systems to identify and help curb the development and spread of AMR. The results from this study suggest that cefixime and ceftriaxone, the drugs for empiric treatment of gonorrhea in Ghana, should be effective against circulating strains. This is likely due to the fact that these drugs are less available to the public; therefore, there is minimal selective pressure to drive cephalosporin resistance at the moment. This notwithstanding, the high prevalence of cephalosporin resistance determinants is a cause for concern with the possibility of emergence and spread of cephalosporin resistance in the near future. Using WGS, we have been able to reveal the unique nature of *N. gonorrhoeae* strains from Ghana and have provided a forecast of the possible state of gonococcal AMR. Ongoing research is needed to continue the tracking of contemporary AMR trends in Ghana and West Africa.

## Data availability statement

The original contributions presented in the study are included in the article/Supplementary material, further inquiries can be directed to the corresponding authors.

## Ethics statement

The study protocol was approved by the Naval Medical Research Command Institutional Review Board in compliance with all applicable Federal regulations governing the protection of human subjects. Research data were derived from an approved Naval Medical Research Command, Institutional Review Board protocol, number NAMRU3.PJT.2019.0007.

## Author contributions

BA, SD, TS, AF, AL, MW, and NA: conceptualization. BA: writing–original draft. HD, KO, JY, and NA: sampling. BA, SK, CY, KO, JY, GB-S: whole genome sequencing. BA and SC: bioinformatics analysis and data interpretation. BA, EB, and NA: data curation. BA, SD, HD, SK, CY, EB, KO, JY, GB-S, SKK, BE, SC, CW, TS, HM, AF, AL, MW, and NA: writing–review and editing. BA, CW, TS, AF, and NA: coordination. BA, SD, CW, TS, and NA: funding acquisition. All authors have read and agreed to the published version of the manuscript.

## Funding

This work was supported by funding from the Armed Forces Health Surveillance Division, Global Emerging Infections Surveillance (GEIS) Branch, ProMIS ID P0137_20_N3_02.01 and a WACCBIP-World Bank ACE Masters/PhD fellowship (ACE02-WACCBIP: Awandare).

## Conflict of interest

The authors declare that the research was conducted in the absence of any commercial or financial relationships that could be construed as a potential conflict of interest.

## Publisher’s note

All claims expressed in this article are solely those of the authors and do not necessarily represent those of their affiliated organizations, or those of the publisher, the editors and the reviewers. Any product that may be evaluated in this article, or claim that may be made by its manufacturer, is not guaranteed or endorsed by the publisher.

## Footnotes

^1^ https://www.bioinformatics.babraham.ac.uk/projects/fastqc/

^2^ https://card.mcmaster.ca/home

^3^ https://card.mcmaster.ca/analyze/rgi

^4^ https://pubmlst.org/organisms/neisseria-spp

^5^ https://pubmlst.org/
