# Supplementary material for: Whole genome analysis and antimicrobial resistance of Neisseria gonorrhoeae isolates from Ghana
Source: Front Microbiol. 2023 Jun 29;14:1163450. doi: 10.3389/fmicb.2023.1163450 (PMC10339232; doi:10.3389/fmicb.2023.1163450)
Supplement: Supplementary file 1 [file Table_1.docx]

**Supplementary 1:** Breakpoints for Antimicrobial Susceptibility Testing.

| Antimicrobial agent | Disc content | MIC Interpretive Standard  µg/ml | | |
| --- | --- | --- | --- | --- |
|  |  | **R** | **I** | **S** |
| Cefixime*** | 5ug | ≥0.25 | - | - |
| Ceftriaxone*** | 30ug | ≥0.125 | - | - |
| Ciprofloxacin | 5ug | ≥1.0 | 0.125-0.5 | ≤0.06 |
| Tetracycline | 30ug | ≥2.0 | 0.5-1.0 | ≤0.25 |
| Spectinomycin | 100ug | ≥128 | 64.0 | ≤32.0 |
| Penicillin | 10units | ≥2.0 | 0.125-1.0 | ≤0.06 |
| Azithromycin*** | 15ug | ≥2.0 |  |  |

CLSI 2018 breakpoints were used for penicillin, tetracycline, spectinomycin and ciprofloxacin. GISP breakpoints (alert values) were used for azithromycin, cefixime, and ceftriaxone for which CLSI resistance breakpoints are not established.

***** No resistance breakpoint available (R represents reduced-susceptibility)

S-susceptible, I-Intermediate, R-resistant/reduced-susceptibility.

CLSI- Clinical and Laboratory Standards Institute.

GISP- Gonococcal Isolate Surveillance Project.
